# The Incremental Yield of CMA over Karyotype in Isolated Absent/Hypoplastic Nasal Bone—A Systematic Review and Meta-Analysis

**DOI:** 10.3390/diagnostics16142235

**Published:** 2026-07-17

**Authors:** Ioakeim Sapantzoglou, Angeliki Rouvali, Alexandros Psarris, Konstantinos Tasias, Maria Ioanna Chatziioannou, Afroditi Pegkou, Zacharias Fasoulakis, Dimitrios Papageorgiou, Marianna Theodora, George Daskalakis, Panagiotis Antsaklis

**Affiliations:** 1 Department of Obstetrics and Gynecology, General Hospital Alexandra, National and Kapodistrian University of Athens, Lourou 4-2, 15238 Athens, Greece; 2 Department of Gynecology, Athens Naval and Veterans Hospital, 11521 Athens, Greece

**Keywords:** fetal nasal bone, hypoplastic fetal nasal bone, absent fetal nasal bone, prenatal assessment, array comparative genomic hybridization, copy number variation

## Abstract

**Background/Objectives**: Absent or hypoplastic nasal bone is strongly associated with Trisomy 21 and other common aneuploidies. Nevertheless, there is a growing body of literature that has associated absence or hypoplasia of the nasal bone with underlying genetic aberrations, other than Trisomy 21. While karyotyping effectively identifies substantial structural mutations, it is limited by its inability to detect submicroscopic copy number variants, thereby constraining the identification of pathological submicroscopic DNA gains or losses. The main objective of our study was to conduct a systematic literature review and a meta-analysis to evaluate the incremental yield of chromosomal microarray analysis compared to karyotyping in cases of isolated absence/hypoplasia of the fetal nasal bone. **Methods**: Our review was designed according to the PRISMA guidelines. It included all observational studies that reported the results of CMA testing in fetuses diagnosed with absent or hypoplastic nasal bone without additional structural abnormalities or findings that would not qualify as structural abnormalities (soft signs) (isolated absent/hypoplastic nasal bone). **Results**: The study included 15 studies with a total of 1328 cases of affected fetuses that met the inclusion criteria for analysis. Combined data from these studies revealed an overall 3% incremental yield of CMA over karyotyping (95% CI 1–4%, I^2^ = 62%) in isolated cases. **Conclusions**: Our findings may be beneficial in clinical practice to provide management strategies and counsel couples, personalizing, as such, patient care and assisting clinicians when encountering this prevalent clinical entity.

## 1. Introduction

The association of absent or hypoplastic nasal bone with Trisomy 21 and other chromosomal abnormalities is well established by several large-scale studies [1,2,3] and its implementation in the first-trimester screening led to a reduction in the false positive rate without causing any impact on the detection rate of the common aneuploidies [4,5]. When the detection of absent or hypoplastic nasal bone takes place in the second trimester, data derived from both metanalysis [6,7] and observational studies of women with homogenous ethnic origin [8,9] have demonstrated an underlying high risk for Down syndrome. In view of the above findings, several authors and guidelines suggest further assessment of the genetic background of the fetus, either through cell-free fetal DNA (cffDNA) or amniocentesis [10,11].

Over the past years and given the emergence and extended use of chromosomal microarray analysis (CMA), which is a molecular approach that identifies submicroscopic copy number variants (CNV) with a resolution of 10 Kb or more, several study groups have evaluated the potential association of isolated and non-isolated fetal nasal bone absence or hypoplasia with underlying genetic aberrations, other than the common aneuploidies [12,13,14]. In this view, while karyotyping effectively identifies substantial structural mutations, polyploidies, and aneuploidies, it is limited by its inability to detect copy number variants, thereby constraining the identification of pathological submicroscopic DNA (deoxyribonucleic acid) gains or losses [15].

As such, the aim of our study is to assess the incremental yield of CMA over standard karyotyping in fetuses with absent or hypoplastic nasal bone without any additional structural abnormalities or soft markers.

## 2. Materials and Methods

This systematic review and meta-analysis was conducted in accordance with the Preferred Reporting Items for Systematic Reviews and Meta-Analyses (PRISMA) standards and the MOOSE standards for Meta-Analyses and Systematic Reviews of Observational Studies. This review has been recorded in the PROSPERO international database for systematic reviews (reference: CRD420251241819).

### 2.1. Eligibility Criteria

This systematic review encompassed all observational studies (prospective/retrospective cohort, case–control, nested case–control, and cross-sectional) that presented results of CMA testing in fetuses diagnosed with absent or hypoplastic nasal bone, devoid of additional structural abnormalities or findings that do not constitute structural abnormalities (soft markers) (isolated absent/hypoplastic nasal bone). Case reports, limited case series, editorial correspondence, animal research, and review papers were excluded. Conference proceedings and abstracts were intended to be omitted due to their deficiencies in important information needed to judge the quality of the evidence and the limits of the study.

### 2.2. Information Sources and Search Strategy

**Figure 1:**
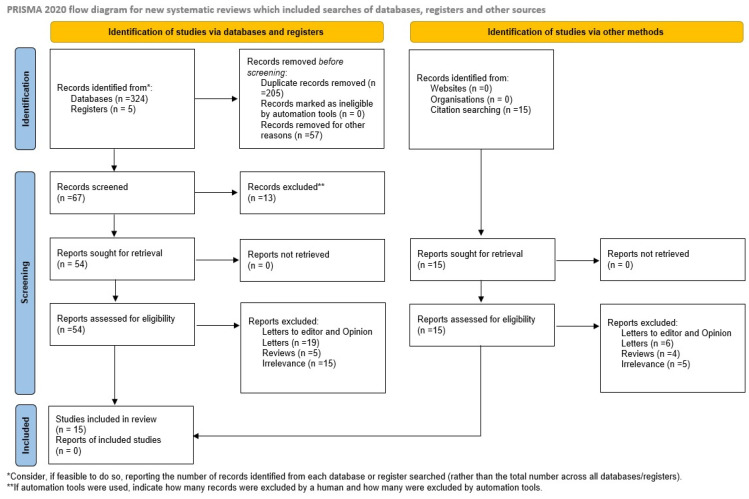
Search strategy [16].

The Medline (1966–2025), Scopus (2004–2025), Clinicaltrials.gov (2008–2025), EMBASE (1980–2025), Cochrane Central Register of Controlled Trials CENTRAL (1999–2025), and Google Scholar (2004–2025) databases were utilized in our initial search, in conjunction with the reference lists of electronically accessed full-text articles. The date of our most recent search was established as 30 November 2025. Our search strategy included the text words “fetal nasal bone” or “hypoplastic fetal nasal bone” or “absent fetal nasal bone” or “fetal assessment” or ‘‘prenatal assessment’’ and “array comparative genomic hybridization” or “copy number variation” and is briefly presented in Figure 1. The main search algorithm was as follows: (“fetal nasal bone” OR “hypoplastic fetal nasal bone” OR “absent fetal nasal bone” OR “fetal assessment” OR ‘‘prenatal assessment’’) AND (“array comparative genomic hybridization” OR ‘aCGH’ OR “copy number variation”). The search yielded 344 potentially relevant studies; however, 329 were removed because they were irrelevant, reviews, opinion letters, or letters to the editor, or because data retrieval from the authors was unsuccessful. Consequently, a total of 15 peer-reviewed articles were deemed suitable for inclusion in our systematic review and the present meta-analysis.

### 2.3. Study Selection

The study selection procedure had three sequential stages. Initially, the titles and abstracts of all electronic papers were evaluated to determine their potential eligibility. Consequently, all papers that satisfied or were anticipated to satisfy the eligibility criteria were obtained in full text. Ultimately, all observational studies, including prospective and retrospective, that presented the outcomes of CMA testing in fetuses identified with isolated absent or hypoplastic nasal bone were considered eligible. Two authors conducted the study selection independently, resolving any potential inconsistencies through consensus. (Figure 1).

### 2.4. Data Collection

The subsequent data were obtained from each included study by three independent investigators (I.S., A.R., D.P.): first author’s name, publication year, study design, recruitment period, inclusion and exclusion criteria, definition of absent/hypoplastic nasal bone, timing of detection, number of women included in each study, criteria for CMA performance, and type of CMA employed.

When critical data were absent, efforts were initiated to reach out to the associated authors. Data extraction was conducted by three investigators (I.S., A.R., D.P.), with any potential disputes settled through consensus or by discussion among all authors. The methodological characteristics of the included studies are illustrated in Table 1.

### 2.5. Quality Assessment

The methodological qualities of the included studies was evaluated by two independent reviewers utilizing the QUADAS-2 (Quality Assessment of Diagnostic Accuracy Studies-2) tool, which assesses patient selection methodology, the indexed test, the reference standard for that test, and the flow and timing of the test/study. A third author provided a final consensus when the two authors did not agree (Table 2). The overall risk was determined based on the recommendations provided by the QUADAS-2 group.

### 2.6. Data Synthesis

The incremental yield (risk difference) of CMA was defined as the yield beyond karyotyping for each prenatal series. The incremental yield was calculated as the ratio of undetected aberrant results by karyotyping (CNV < 10 Mb at microarray analysis) to the total number of cases with a normal karyotype. Variants of uncertain significance were excluded from this study.

Risk differences were consolidated using inverse variance weighting to estimate the overall and stratified CMA incremental yield through RStudio (RStudio version 4.6.0, RStudio Inc., Boston, MA, USA). Subsequently, corresponding forest plot graphs were generated. Confidence intervals (CIs) were computed. The Higgins I^2^ test was employed to evaluate statistical heterogeneity. Due to the limited statistical power of heterogeneity tests, we established statistically significant heterogeneity as a Cochran Q test with *p* < 0.1 or I^2^ > 30%. A random-effects model was utilized due to substantial heterogeneity.

To evaluate the robustness of statistically significant findings, we calculated the Fragility Index (FI) for each dichotomous meta-analysis. The FI represents the minimum number of event status modifications (i.e., converting a non-event to an event, or vice versa, across included studies) required to change a statistically significant pooled effect (*p* < 0.05) to a non-significant one (*p* ≥ 0.05). A small FI indicates fragile evidence, meaning statistical significance depends on very few outcome changes.

To quantify the statistical power of our meta-analytic comparison between CMA and conventional karyotyping, we performed a post hoc power calculation for a two-sample comparison of proportions. The analysis was based on the total number of fetuses included in the meta-analysis (*n* = 1328 evaluated with each method), assuming a baseline rate of clinically significant chromosomal abnormality of 0% with conventional karyotyping and an absolute risk difference corresponding to the observed incremental diagnostic yield of CMA. Power was estimated using a two-sided test for the difference in proportions at α = 0.05, with equal group sizes (n_0_ = n_1_ = 1328) and event probabilities p_0_ and p_1_ for conventional karyotyping and CMA, respectively. In addition, we estimated the minimal absolute risk difference that the available sample size could detect with 80% power at α = 0.05, to contextualize the sensitivity of the meta-analysis to smaller incremental diagnostic yields.

## 3. Results

Our search identified 344 potentially relevant studies, but 329 were excluded after reviewing the titles and the abstracts, and after the exclusion of non-relevant articles, case reports, opinion letter, reviews and letters to the editor. Overall, 15 studies were included in the present systematic review (13 retrospective and 2 prospective cohort studies) that enrolled a total of 1328 cases of patients [12,13,14,17,18,19,20,21,22,23,24,25,26,27,28]. The search strategy is briefly presented in Figure 1.

The methodological characteristics of the included studies are presented in Table 1 and include the inclusion and exclusion criteria, absent/hypoplastic nasal bone definition, timing of detection, number of women included in each study, the inclusion criteria for the performance of CMA and the type of CMA utilized. The participant recruitment periods of the included studies ranged from 2004 to 2022, and the specific recruitment period of each study is depicted in Table 1.

Absent/hypoplastic nasal bone definition was provided in 13 studies while the remaining 2 studies did not provide any specific diagnostic criterion [12,24]. Absent nasal bone was defined unanimously as no visualization in any of the appropriate planes. Eight studies defined hypoplastic nasal bone as shorter than the 2.5th centile for the gestational age [12,14,17,18,20,25,27,28], one study defined it as shorter than 2.5 mm [22], one study defined it as absence of the clear second line of the nose [18], two studies defined it as appearing short or hypoechoic or dysplastic without providing specific cut-off or ultrasound parameters [21,26] and one first trimester study defined it the absence of the three distinct lines at the level of nasal bone (as suggested by Cicero et al. [1]) [23].

CMA was performed after a normal result at karyotyping in two studies [12,27], in selected cases of included women according to clinical indication, gestational age and the time period of examination in one study [20], in selected cases of included women with nuchal translucency < 99th centile in one study [23] and in all included women in 10 studies [13,14,17,18,19,21,22,25,26,28]. One study did not provide any data on the basis on which CMA was performed in the sample [24].

### Synthesis of Results

**Figure 2:**
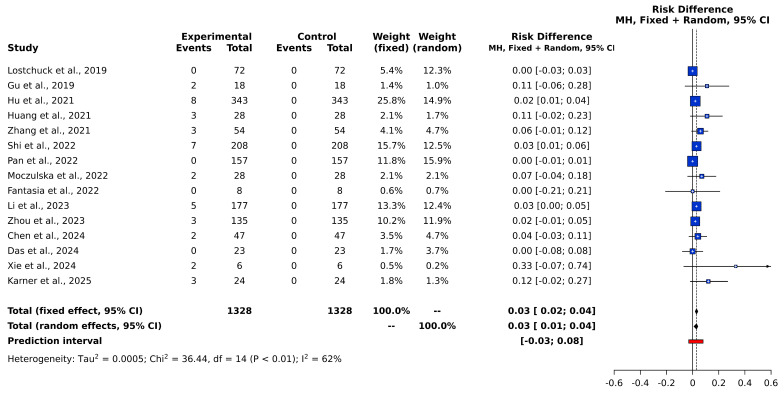
Forest plot of incremental yield by chromosomal microarray analysis (CMA) over karyotyping in isolated nasal bone absence or hypoplasia with 95% confidence intervals (CIs) and weighted pooled summary statistics using a bivariate random-effect model. Forest plot analysis: vertical line = “no difference” point between the two groups; blue squares = incremental yield of CMA over karyotyping of individual studies; diamond = pooled incremental yield of CMA over karyotyping and 95% CI for all studies; horizontal black lines = 95% CI; horizontal red line = prediction intervals [12,13,14,17,18,19,20,21,22,23,24,25,26,27,28].

The forest plot of the 15 included studies and the pooled results from the meta-analysis in cases of isolated absent/hypoplastic nasal bone are depicted in Figure 2. The pooled data from the reviewed studies show an overall 3% incremental yield of CMA over karyotyping (95% CI 1–4%, I^2^ = 62%). The observed incremental yield for each single study ranged from 0 to 33% in isolated cases.

The clinically significant submicroscopic CNVs detected in fetuses with absent or hypoplastic nasal bone are presented in Table 3, whenever applicable, the association of pathogenic or likely pathogenic CNVs with either hypoplastic or absent nasal bone was demonstrated.

**Figure 3:**
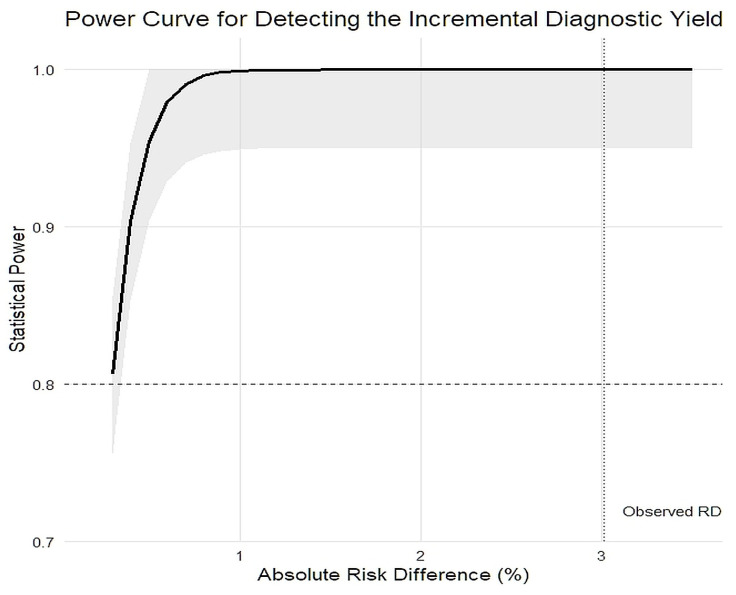
Depiction of the statistical power to detect varying absolute risk differences (incremental diagnostic yields) between CMA and conventional karyotyping across the total sample size included in the meta-analysis (*n* = 1328 per method). Solid black line: estimated power; shaded band: 95% confidence intervals region; horizontal dashed line: 80% power threshold; vertical dotted line: observed pooled risk difference of 1.9%. The curve depicts that even modest diagnostic yields (≈0.6%) reach adequate power, while the observed yield lies in a region of >99% power, consistent with the high sensitivity of the analysis despite the rarity of abnormal CMA findings.

Using the summary effect estimate and the total sample size of 1328 fetuses per method, the post hoc power of the meta-analysis to detect an absolute risk difference of 1.9% at α = 0.05 was essentially >99.9%. Even for a more modest absolute increase in diagnostic yield of 1.0%, the available sample size would still provide approximately 96% power, and an absolute risk difference of only 0.6% would be detectable with around 80% power. These calculations indicate that the study was statistically well-powered to detect the observed incremental diagnostic value of CMA over conventional karyotyping in fetuses with absent or hypoplastic nasal bone and a normal conventional karyotype (Figure 3).

**Figure 4:**
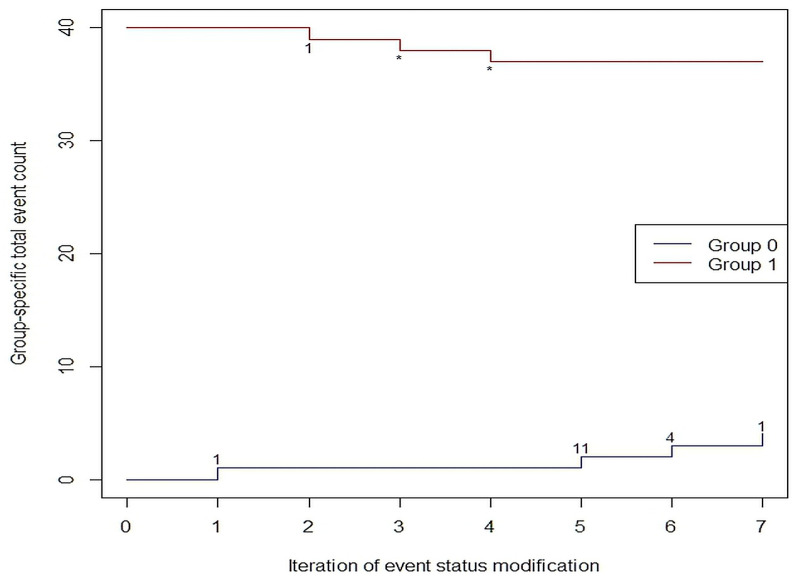
Illustration of the stepwise modification of event counts used to calculate the fragility index (FI). Statistical significance was lost after seven event changes (FI = 7; FQ = 0.3%), indicating that the pooled 1.9% incremental diagnostic yield of CMA depends on a small number of abnormal findings. This fragility reflects the rarity of pathogenic CMA results rather than instability of the analysis. Asterisk (*) indicates that the event-status modification was applied to the same study and group as in the preceding iteration; numbers identify the studies in which event counts were modified.

Nevertheless, it should be mentioned that the fragility index (FI) was 7, meaning that changing the classification of only seven fetuses (i.e., recoding seven CMA-positive cases as negative, or equivalently adding seven events to the conventionally tested group) would render the pooled RD non-significant. The corresponding fragility quotient (FQ) was 0.3%, indicating that less than 0.3% of all participants would need to have a different outcome for the observed statistical significance to be lost (Figure 4).

## 4. Discussion

The study demonstrated an overall of 3% incremental yield of CMA over karyotyping (95% CI 1–4%, I^2^ = 62%) in cases with absent or hypoplastic nasal bone without any additional findings.

To our knowledge, this meta-analysis encompasses and summarizes data from all the available studies up to date that have assessed the additive diagnostic yield of CMA in karyotypic normal fetuses with isolated fetal nasal bone absence or hypoplasia.

It is generally recognized that the nasal bone is absent, hypoplastic or not ossified in the majority of fetuses with Trisomy 21 and its correct assessment and implementation in the first trimester screening for Down syndrome has improved its performance [29]. However, the association of nasal bone hypoplasia/absence with additional genetic abnormalities remains undetermined, even though it has been investigated by several cohort studies [17,18,19,20,21,22,23,24,25,26,27,28]. In view of the inconclusive association with underlying pathogenic copy number variations, the recently published Society for Maternal–Fetal Medicine (SMFM) Consult Series have advised that pregnant individuals without prior aneuploidy screening and an isolated absent or hypoplastic nasal bone should be solely counselled for assessment of the risk of Trisomy 21 with potential consideration of non-invasive aneuploidy screening (cffDNA) while no further testing is deemed necessary if cffDNA demonstrates low risk for Trisomy 21 [10]. Similarly, no mention of the potential investigation of submicroscopic genetic aberrations is made in other guidelines should isolated nasal bone absence or hypoplasia be detected [30,31].

However, the extensive application of molecular genetic diagnostics throughout the antenatal period has expedited research on the efficacy of CMA in both isolated and non-isolated cases of nasal bone absence or hypoplasia. The data regarding the incremental yield of CMA in truly isolated cases of nasal bone absence or hypoplasia have been inconsistent so far. It is well established that non-isolated cases carry an increased risk of both chromosomal and submicroscopic abnormalities [9,32]. This was further emphasized by several of the included studies in which CMA resulted in submicroscopic pathogenic or likely pathogenic CNVs only when nasal bone absence or hypoplasia was accompanied by additional second-trimester soft markers [17] or when first-trimester nuchal translucency was measuring above the 99th centile [23]. Similarly, Lostchuck et al. demonstrated in their cohort that there were no cases of abnormal copy-number variations in fetuses exhibiting second-trimester isolated hypoplastic nasal bone while submicroscopic pathogenic CNVs were observed in 30% of non-isolated cases [24]. However, modern research has produced different results. Xie et al. demonstrated that among 14 fetuses with isolated nasal bone absence or hypoplasia, 8 were affected by a chromosomal abnormality, with Trisomy 21 being the most prevalent, and 2 were affected by a clinically significant CNV [28]. However, no data regarding the first-trimester screening was provided, a fact that might have had an impact on the underlying risk of genetic conditions. Furthermore, Shi et al. investigated 208 fetuses with isolated nasal bone abnormality (after the exclusion of fetuses with increased NT, other soft signs or structural abnormalities) revealing seven cases with a submicroscopic pathogenic CNV [14]. Similarly, in the study of Li et al., CMA revealed five cases of pathogenic CNVs after conventional karyotyping in isolated cases of low-risk women who have undergone either maternal serological screening or non-invasive prenatal testing (NIPT) [19]. In accordance with the above findings, Gu et al. demonstrated that, in the absence of a normal nasal in the second trimester, CMA may provide an incremental yield by identifying abnormal submicroscopic CNVs, even when the finding is isolated and the screening test for fetal aneuploidies indicates a low risk. Such a finding is in accordance with previous reports, such as the results demonstrated by Hu et al., according to which eight cases of submicroscopic pathogenic or likely pathogenic CNVs were detected by CMA in a cohort of 343 women after excluding fetuses with NT of more than 3 mm, structural abnormalities or a positive NIPT result [22].

To our knowledge, this is the first metanalysis that summarized all the available evidence and investigated the incremental yield of CMA over karyotyping in fetuses with isolated absent or hypoplastic nasal bone. Our study analyzed data from 15 studies and included a total of 1328 fetuses that underwent CMA investigation after a normal karyotype analysis. The main strengths of this study were its sample size and the fact that the included studies were meticulously investigated to avoid including non-isolated cases of absent or hypoplastic nasal bone in the final analysis, so our results were as concrete as possible. Furthermore, the definition of the absence of fetal nasal bone was homogenous among included studies.

However, there are certain limitations to be noted. There is heterogeneity in the definition of hypoplastic nasal bone since five different diagnostic modules were adopted across the included studies. In addition, two studies did not report the definition used. Furthermore, the recruitment period of several studies spans over a period of more than 10 years and, as such, older ultrasound technology might have had an impact on the detection rates of nasal hypoplasia or absence [20,25]. Another methodological concern lies on the fact that different CMA platforms were used, a fact that may affect the subsequent detection rate. However, given that most of the included studies provided no additional information on the CMA type used, a subgroup analysis could not be performed. Furthermore, in six studies, the detection of absent and/or hypoplastic nasal bone was undertaken in either the first or second trimester. However, only two studies provided specific data regarding the incremental yield of CMA when the detection occurred in the first trimester [14,19]. As such, a subgroup analysis was omitted. Furthermore, another methodological issue that was noted is the sequencing on which CMA was conducted, since CMA was performed in all included women in 10 studies [13,14,17,18,19,21,22,25,26,28], after a normal karyotype result in two studies [12,26] and in selected cases based on clinical indication in others [20,23]. Such an approach may influence the incremental yield rates given that the performance of CMA in all cases may demonstrate different yield compared to when CMA is performed only after a normal conventional karyotyping result. Additionally, the retrospective study design of most of the included studies is known to be prone to inherent bias, while a number of them included diverse ethnic populations [23,25]. Such ethnic diversity may inappropriately increase the number of fetuses that screen positive, given the fact that nasal bone length has been documented across several ethnic groups and is demonstrated to be substantially shorter in certain populations, particularly in fetuses of Afro-Caribbean and Asian descent [8,33,34]. Furthermore, an effort was made to fully isolate cases of fetal nasal bone absence or hypoplasia and, as such, we excluded, wherever that was applicable, cases with increased first-trimester nuchal translucency measurements, those with additional structural anomalies or soft signs, and pregnancies that were considered high-risk after the first-trimester screening or a positive cffDNA result. Nevertheless, a limited number of the included studies [21,24,26] did not provide any first-trimester data or screening results, a fact that could potentially have an impact on our sample and our results. Finally, it should be underlined that, as expected, the incremental yield of CMA was based on the pregnancies that eventually underwent invasive testing and not on all cases with hypoplastic/absent nasal bone, a fact that could potentially lead to an increase in the rate of pathogenic submicroscopic CNVs.

Nevertheless, the results of our study underline a potential association between isolated absent or hypoplastic nasal bone with submicroscopic genetic aberrations and, as already stated by other authors [26,28], these findings support consideration of CMA should a nasal bone abnormality be detected but warrant prospective evaluation.

## 5. Conclusions

Our study demonstrated an overall 3% incremental yield of CMA over karyotyping in fetuses with absent or hypoplastic nasal bone, without any additional findings. These findings may be beneficial in clinical practice to provide management strategies and counsel couples, personalizing, as such, patient care and assisting clinicians when encountering this prevalent clinical entity.

## Figures and Tables

**Table 1:** Methodological characteristics of the included studies.

Author, Year	Type of Study	Recruitment Period	Inclusion Criteria	Exclusion Criteria	Absent/Hypoplastic Nasal Bone Definition	Timing of Detection	Number of Included Women	Inclusion Criteria for CMA	CMA Type
Zhou et al., 2023 [12]	Retrospective cohort	NR	Fetuses with isolated and non-isolated ultrasound soft markers Invasive prenatal diagnosis assessment for karyotype and/or CMA) by categorizing into two groups	Presence of other structural abnormalities Multiple pregnancies Increased NT High risk antenatal serum screening/NIPT Missing data	NR	11–35 weeks of gestation	358 cases	Fetuses with a normal karyotype	Affymetrix CytoScan HD
Zhang et al., 2021 [13]	Retrospective cohort	November 2018–March 2020	Isolated fetal nasal bone absence/hypoplasia in the second trimester	Missing data	Absent NB: not visualized on any appropriate view. Hypoplastic NB: nasal bone length below the 2.5th percentile	Second trimester	55 cases	All included patients	Affymetrix CytoScan 750 K Array chip, DNA (250 ng)
Shi et al., 2022 [14]	Retrospective cohort	Jan 2015-April 2021	Isolated fetal nasal bone absence/hypoplasia	Presence of other structural abnormalities Markers for chromosomal abnormalities Missing data	Absent NB: not visualized on any appropriate view (sagittal, transverse and coronal sections) Hypoplastic NB: nasal bone length below the 2.5th percentile of local population charts	First and/or second trimester	221 cases	All included patients	Affymetrix CytoScan 750 K Array
Pan et al., 2022 [17]	Retrospective cohort	May 2017–December 2021	Isolated and non-isolated fetal nasal bone absence/hypoplasia in the second trimester	Multiple pregnancies Missing data	Absent NB: not visualized on any appropriate view. Hypoplastic NB: nasal bone length below the 2.5th percentile	14–27 + 6 weeks of gestation	351 cases	All included patients	Array-based comparative genomic hybridization was performed using a customized Fetal DNA Chip (Agilent Technologies, Santa Clara, CA, USA)
Moczulska et al., 2022 [18]	Retrospective cohort	2016–2021	Isolated and non-isolated fetal nasal bone absence/hypoplasia in the second trimester	Fetuses with trisomy 21, 18 and 13 Missing data	Absent NB: not visualized on any appropriate view. Hypoplastic NB: nasal bone length below the 2.5th percentile	Second trimester	60 cases	All included patients	GenetiSure Pre-Screen Kit 8 × 60 K (Agilent)
Li et al., 2023 [19]	Retrospective cohort	May 2017–December 2021	Isolated and non-isolated fetal nasal bone absence/hypoplasia Performance of an invasive prenatal assessment by CMA and/or karyotyping	Missing data	Absent or hypoplasia of the nasal bone corresponds to the absence of clear second line of the nose	First, second and third trimester	320 cases	All included patients	Cytoscan 750 K array Affymetrix
Karner et al., 2025 [20]	Retrospective cohort	2004–2022	Isolated and non-isolated fetal nasal bone absence/hypoplasia	Patients who were lost to follow-up Multiple pregnancies Normal fetal NB in the second trimester Missing data	Absent NB: not visualized on any appropriate view. Hypoplastic NB: nasal bone length below the 2.5th percentile	First and second trimester	149 ses	Selected cases of included patients according to clinical indication, GA and the time period of examination	NR
Huang et al., 2021 [21]	Retrospective cohort	December 2016–December 2018	Isolated and non-isolated fetal nasal bone absence/hypoplasia Performance of an invasive prenatal assessment by CMA and/or karyotyping	Missing data	Absence or dysplasia of the NB on the mid–sagittal plane of the second trimester scan	18–36 weeks of gestation	84 cases	All included patients	Affymetrix CytoScan 750 K GeneChip Platform
Hu et al., 2021 [22]	Prospective cohort	September 2014–August 2019	Presence of other soft markers	NT ≥ 3.0 mm Positive NIPT results for aneuploidies Presence of other structural abnormalities Missing data	Absent NB: not visualized on any appropriate view Hypoplastic NB: nasal bone length shorter than 2.5 mm	Second trimester	2466 cases	All included patients	SNP array with the CytoScan 750 K Array
Fantasia et al., 2022 [23]	Retrospective cohort	2013–2018	Absent NB in the first trimester Persistence of absent/hypoplastic NB at subsequent scans Postnatal 1 year follow-up	First trimester scan for pregnancy dating only NB visualized at subsequent scans with normal dimensions Second-trimester routine scan and Loss of postnatal follow-up Missing data	Three distinct lines are seen at the level of the fetal nose	11 + 0 to 13 + 6 weeks of gestation	86 cases	Selected cases of included patients if NT < 99th centile	Array comparative genomic hybridization or high-density single nucleotide polymorphism array techniques
Lostchuck et al., 2019 [24]	Secondary analysis (Retrospective cohort)	2012–2016	Hypoplastic NB in the second trimester	Missing data	NR	18–22 weeks of gestation	127 cases	NR	NR
Gu et al., 2019 [25]	Retrospective cohort	January 2006–December 2015	Singleton and dichorionic twin pregnancies with hypoplastic nasal bone Performance of an invasive prenatal assessment by CMA and/or karyotyping or newborn examination data	Genetic or newborn examination information not available Missing data	Hypoplastic NB: absence or nasal bone length below the 2.5th percentile	16–37 weeks of gestation	58 cases pre-CMA era 60 cases CMA era	All included patients	Affymetrix CytoScan 750 K or Illumina HumanCytoSNP-12
Chen et al., 2024 [26]	Retrospective cohort	NR	Isolated and non-isolated fetal nasal bone absence/hypoplasia	Missing data	Absent NB: not visualized on any appropriate view Hypoplastic NB: nasal bone appears short or hypoechoic	16–24 weeks of gestation	94 cases	All included patients	Affymetrix Cytoscan 750 K chip
Das et al., 2024 [27]	Prospective cohort	NR	Isolated and non-isolated fetal nasal bone absence/hypoplasia on first or second trimester	Missing data	Absent NB: not visualized on any appropriate view Hypoplastic NB: nasal bone length below the 2.5th percentile	11–22 weeks of gestation	47 cases	Fetuses with a normal karyotype	NR
Xie et al., 2024 [28]	Retrospective cohort	May 2017–May 2022	Isolated and non-isolated fetal nasal bone absence/hypoplasia Performance of an invasive prenatal assessment by CMA and/or karyotyping	Missing data	Absent NB: not visualized on any appropriate view Hypoplastic NB: nasal bone length below the 2.5th percentile	12–34 weeks of gestation	333 cases	All included patients	CytoScan 750 K gene chip detection platform

Abbreviations: NB: nasal bone, NT: nuchal translucency, CMA: chromosomal microarray analysis, SNP: single nucleotide polymorphism, NR: not reported.

**Table 2:** Quality assessment summary of the included studies using the Quality Assessment tool for Diagnostic Accuracy Studies (QUADAS-2) checklist.

	Patient Selection					Index Test(s)				Reference Standard				Flow and Timing				
Author, Year	Was a Consecutive or Random Sample of Patients Enrolled?	Was a Case-Control Design Avoided?	Did the Study Avoid Inappropriate Exclusions?	Risk of Bias	Concerns Regarding Applicability	Were the Index Test Results Interpreted Without Knowledge of the Results of the Reference Standard?	If a Threshold Was Used, Was It Pre-Specified?	Risk of Bias	Concerns Regarding Applicability	Is the Reference Standard Likely to Correctly Classify the Target Condition?	Were the Reference Standard Results Interpreted Without Knowledge of the Results of the Index Test?	Risk of Bias	Concerns Regarding Applicability	Was There an Appropriate Interval Between Index Test(s) and Reference Standard?	Did All Patients Receive a Reference Standard?	Did Patients Receive the Same Reference Standard?	Were All Patients Included in the Analysis?	Risk of Bias
Lostchuck et al., 2019 [24]	no	yes	unclear	unclear	high	no	yes	low	low	yes	no	low	low	no	yes	yes	yes	low
Gu et al., 2019 [25]	no	yes	yes	no	low	no	yes	low	low	yes	no	low	low	no	yes	yes	yes	low
Hu et al., 2021 [22]	no	yes	yes	no	low	no	yes	low	low	yes	no	low	low	no	yes	yes	yes	low
Huang et al., 2021 [21]	no	yes	unclear	no	low	no	yes	low	low	yes	no	low	low	no	yes	no	yes	low
Zhang et al., 2021 [13]	no	yes	yes	no	low	no	yes	low	low	yes	no	low	low	no	yes	yes	yes	low
Shi et al., 2022 [14]	no	yes	yes	no	low	no	yes	low	low	yes	no	low	low	no	yes	yes	yes	low
Pan et al., 2022 [17]	no	yes	yes	no	low	no	yes	low	low	yes	no	low	low	no	yes	yes	yes	low
Moczulska et al., 2022 [18]	no	yes	unclear	yes	high	no	yes	low	low	yes	no	low	low	no	yes	yes	yes	low
Fantasia et al., 2022 [23]	no	yes	unclear	unclear	unclear	no	yes	low	low	yes	no	low	low	no	yes	yes	yes	low
Li et al., 2023 [19]	no	yes	yes	no	low	no	yes	low	low	yes	no	low	low	no	yes	yes	yes	low
Zhou et al., 2023 [12]	no	yes	unclear	no	low	no	yes	low	low	yes	no	low	low	no	yes	yes	yes	low
Chen et al., 2024 [26]	no	yes	yes	no	low	no	yes	low	low	yes	no	low	low	no	yes	yes	yes	low
Das et al., 2024 [27]	no	yes	yes	no	low	no	yes	low	low	yes	no	low	low	no	yes	yes	yes	low
Xie et al., 2024 [28]	no	yes	yes	no	low	no	yes	low	low	yes	no	low	low	no	yes	no	no	low
Karner et al., 2025 [20]	no	yes	unclear	no	low	no	yes	low	low	yes	no	low	low	no	yes	no	yes	low

**Table 3:** Pathogenic and likely pathogenic CNVs identified by CMA analysis in fetuses with isolated fetal nasal bone absence or hypoplasia and normal karyotype.

Author, Year	Nasal Bone	CNVs	Fragment Size
Zhou et al., 2023 [12]	Absent NB	Deletion: 18p11.32p11.31 Duplication: 22q11.21	2.53 Mb 2.82 Mb
	Hypoplastic NB	Deletion: Xp22.33	1.95 Mb
Zhang et al., 2021 [13]	Absent NB	Deletion 10q11.22	5.7 Mb
	Hypoplastic NB	Duplication 1q21.1q21.2 Deletion 4p16.3p16.1	1.3 Mb 7.6 Mb
Shi et al., 2022 [14]	Absent NB	Deletion Xp22.33 or Yp11.32 Deletion 14q22.1q22.3 Deletion 6p21.1p12.3 Deletion 1q21.1q21.2	713 kb 3.0 Mb 4.1 Mb 1.3 Mb
	Hypoplastic NB	Duplication Xp22.31 Deletion 1p36.33p36.31 Deletion 16p11.2	1.7 Mb 4.9 Mb 761 kb
Pan et al., 2022 [17]	Hypoplastic NB	N/A	N/A
Moczulska et al., 2022 [18]	Hypoplastic NB	Deletion Xp22.31 Deletion 8p23.3p23.1 and duplication 8.23.1p12 *	1.67 Mb 6.68 Mb
Li et al., 2023 [19]	Absent or Hypoplastic NB	Duplication 16p13.3 Mosaic deletion 13q13.2q34 Duplication 9p24.3p13.1 Deletion 9q31.2q33.2 17p12 *	NR
Karner et al., 2025 [20]	Absent or Hypoplastic NB	Duplication in 5q21.1 Deletion in 17q22 Deletion on chromosome 2	NR
Huang et al., 2021 [21]	Hypoplastic NB	Deletion 15q13.2q13.3 Deletion 16p12.2 Deletion 17p12	2.0 Mb 0.97 Mb 1.4 Mb
Hu et al., 2021 [22]	Absent NB	Duplication 1q21.1 Deletion 7q11.23 (Williams-Beuren Syndrome) Deletion 17p12 Deletion 17p12	2109 Kb 1530 Kb 1383 Kb 1396 Kb
	Hypoplastic NB	Duplication 1q21.1 Duplication 1q21.1 Deletion 1p22.1p21.1 * Deletion 18q21.32q23 *	889 Kb 2686 Kb 8156 Kb 9907 Kb
Fantasia et al., 2022 [23]	Absent NB	N/A	N/A
Lostchuck et al., 2019 [24]	Hypoplastic NB	N/A	N/A
Gu et al., 2019 [25]	Absent NB	Deletion 17p11.2 (Smith Magenis)	N/A
	Hypoplastic NB	Deletion 4p16.3p16.1 and duplication 9p24.3 (Wolf Hirshhorn syndrome)	
Chen et al., 2023 [26]	Absent or Hypoplastic NB	Duplication 16p13.3 Deletion 20p13p12.3	7.7 Mb 3.1 Mb
Das et al., 2024 [27]	Absent or Hypoplastic NB	N/A	N/A
Xie et al., 2023 [28]	Absent NB	17p12	1.4 Mb
	Hypoplastic NB	Deletion 15q13.2q13.3	2.0 Mb

Abbreviations: NB: Nasal bone, N/A: not applicable, NR: not reported. * Likely pathogenic.

## Data Availability

No new data were created or analyzed in this study. Data sharing is not applicable to this article.

## Abbreviations

The following abbreviations are used in this manuscript:

cffDNA	Cell-free fetal DNA
CMA	Chromosomal microarray analysis
DNA	Deoxyribonucleic acid
PRISMA	Preferred Reporting Items for Systematic Review and Meta-Analysis
aCGH	Array Comparative Genomic Hybridization
CNV	Copy Number Variants
QUADAS-2	Quality Assessment of Diagnostic Accuracy Studies-2
